# In Silico Identification of Potential Inhibitors of SARS-CoV-2 Main Protease (M^pro^)

**DOI:** 10.3390/pathogens13100887

**Published:** 2024-10-11

**Authors:** Manuel Alejandro Hernández-Serda, Víctor H. Vázquez-Valadez, Pablo Aguirre-Vidal, Nathan M. Markarian, José L. Medina-Franco, Luis Alfonso Cardenas-Granados, Aldo Yoshio Alarcón-López, Pablo A. Martínez-Soriano, Ana María Velázquez-Sánchez, Rodolfo E. Falfán-Valencia, Enrique Angeles, Levon Abrahamyan

**Affiliations:** 1 Departamento de Ciencias Químicas FES Cuautitlán, Universidad Nacional Autónoma de México (UNAM), Av. 1 de Mayo SN Cuautitlán Izcalli, Mexico City 54750, Mexico; serda@cuautitlan.unam.mx (M.A.H.-S.); yoshalar@gmail.com (A.Y.A.-L.); arturin_sirio@yahoo.com.mx (P.A.M.-S.); velzquezanamara@gmail.com (A.M.V.-S.); angeles@unam.mx (E.A.); 2 Departamento de Ciencias Biológicas FES Cuautitlán, Universidad Nacional Autónoma de México (UNAM), Av. 1 de Mayo SN Cuautitlán Izcalli, Mexico City 54750, Mexico; hugounam83@gmail.com; 3 QSAR Analytics S.A. de C.V. Coatepec 7, Cumbria, Cuautitlán Izcalli, Ciudad de México 54750, Mexico; 4 Laboratorio de Química Medicinal y Teórica FES Cuautitlán, Universidad Nacional Autónoma de México (UNAM), Campo 1 Av. 1 de Mayo SN Cuautitlán Izcalli, Mexico City 54750, Mexico; pyogenes2heli@gmail.com (P.A.-V.); aalfonsocardenas@gmail.com (L.A.C.-G.); rodolfoefvv@gmail.com (R.E.F.-V.); 5 Swine and Poultry Infectious Diseases Research Center (CRIPA), Research Group on Infectious Diseases in Production Animals (GREMIP), Faculty of Veterinary Medicine, University of Montreal, Saint-Hyacinthe, QC J2S 2M2, Canada; nathan.marko.markarian@umontreal.ca; 6 Faculté de Pharmacie, Université de Montréal, Montreal, QC H3T 1J4, Canada; 7 DIFACQUIM Research Group, Department of Pharmacy, School of Chemistry, National Autonomous University of Mexico, Av. Universidad 3000, Ciudad de México 04510, Mexico; medinajl@unam.mx

**Keywords:** SARS-CoV-2, M^pro^, 3CL^pro^, Nsp5, antivirals

## Abstract

The ongoing Coronavirus Disease 19 (COVID-19) pandemic has had a profound impact on the global healthcare system. As the SARS-CoV-2 virus, responsible for this pandemic, continues to spread and develop mutations in its genetic material, new variants of interest (VOIs) and variants of concern (VOCs) are emerging. These outbreaks lead to a decrease in the efficacy of existing treatments such as vaccines or drugs, highlighting the urgency of new therapies for COVID-19. Therefore, in this study, we aimed to identify potential SARS-CoV-2 antivirals using a virtual screening protocol and molecular dynamics simulations. These techniques allowed us to predict the binding affinity of a database of compounds with the virus M^pro^ protein. This in silico approach enabled us to identify twenty-two chemical structures from a public database (QSAR Toolbox Ver 4.5 ) and ten promising molecules from our in-house database. The latter molecules possess advantageous qualities, such as two-step synthesis, cost-effectiveness, and long-lasting physical and chemical stability. Consequently, these molecules can be considered as promising alternatives to combat emerging SARS-CoV-2 variants.

## 1. Introduction

At the end of 2019, numerous cases of a mysterious respiratory disease were reported in Wuhan, China [1]. As time passed, the number of cases and deaths increased, and soon enough, the causative agent was discovered to be a novel betacoronavirus, known today as Severe Acute Respiratory Syndrome coronavirus 2 (SARS-CoV-2) [2]. SARS-CoV-2 has spread throughout the world, leading to the global COVID-19 pandemic [3]. As a countermeasure to slow the spread of SARS-CoV-2, several countries decided to implement lockdown measures, which had significant repercussions on public health, the environment, human psychology, the global socioeconomic situation, and education [4]. Despite these measures, the virus continues to spread, and as of 23 June 2024, there are over 775 million confirmed COVID-19 cumulative cases of infection, of which more than 7 million have resulted in death [5].

Coronaviruses (CoVs) are enveloped positive-strand RNA viruses with the largest known RNA genomes of 30–32 kilobases (kb) [6]. Both animals and humans can be infected with these viruses, which raises the importance of keeping track of their zoonotic potential [7]. There are seven known human coronaviruses, four of which are endemic and generally cause mild to moderate upper respiratory tract symptoms [8]. These include 229-CoV, OC43-CoV, HKU1-CoV, and NL63-CoV [8]. The remaining three CoVs are betacoronaviruses known to cause pandemics in the 21st century, including SARS-CoV, MERS-CoV, and SARS-CoV-2 [9]. At the nucleotide level, SARS-CoV-2 shares approximately 79% sequence identity with SARS-CoV and approximately 50% with MERS-CoV, both of which were responsible for coronaviral pandemics in the 20th century [10]. Phylogenetically, since SARS-CoV-2 is a betacoronavirus, it belongs to the order *Nidovirales*, family *Coronaviridae*, subfamily *Orthocoronavirinae*, genus *Betacoronavirinae*, and subgenus *Sarbecovirus* [11].

The SARS-CoV-2 genome is composed of different open reading frames (ORFs), of which ORF 1a/b occupies two-thirds; the rest are occupied by other ORFs coding for accessory (i.e., ORF3a, ORF6, ORF7, ORF8, and ORF9) and structural (spike, nucleocapsid, membrane, and envelope) proteins [12]. Accessory proteins such as ORF3a, ORF6, ORF7, and ORF8 have various functions, including type IFN-I antagonism and suppression of viral responses [13]. Structural proteins include surface glycoprotein S, envelope protein E, membrane protein M, and nucleocapsid protein N, which are necessary for virus assembly and infectivity [14]. To invade host cells, SARS-CoV-2 uses its spike protein, which allows it to bind to the host cell receptor angiotensin converting enzyme 2 (ACE2); this binding is followed by a conformational change in the S protein, ultimately facilitating the fusion of the viral envelope with the cell membrane [15]. In this way, SARS-CoV-2 releases its genomic RNA into the host cell, which is directly converted to ppa1a and pp1ab, which, in turn, are proteolytically cleaved, giving rise to 10 and 16 proteins, respectively [16], which form the viral replicase transcriptase complex (RTC) [17]. This cleavage is achieved by the action of viral-encoded proteases known as papain-like proteases (PL^pro^, protease domain of Nsp3) and chymotrypsin-like (3CL^pro^) or main protease domains (M^pro^) of Nsp5 [17]. Both the latter, along with the spike protein, are important therapeutic targets [18]. PL^pro^ is the largest nonstructural protein encoded by SARS-CoV-2 and cleaves the viral polyprotein pp1ab to produce Nsp1-3 [19]. However, the homodimeric cysteine protease M^pro^ can proteolytically cleave the pp1ab polyprotein at 11 cleavage sites, which makes it important in the viral replication cycle [20,21]. In its monomeric form, M^pro^ has a molecular weight of 34.21 kDa and comprises three domains: domain I (residues 8–101), domain II (residues 102–184), and domain III (residues 201–303), where domains I and II have an antiparallel β-barrel structure and domain III contains five α-helices arranged into a largely antiparallel globular cluster [22,23]. The catalytic site of M^pro^ is located at the intersection of domains I and II, with a catalytic dyad formed by Cys145 and His41. It is important to note that M^pro^ is less enzymatically active than its dimeric form [22].

Currently, there are many ways to control the COVID-19 pandemic: vaccines [24,25], neutralizing monoclonal antibodies (mAbs) against the spike protein, and the antiviral drugs ritonavir-boosted nirmatrelvir (targeting the M^pro^) [26], molnupiravir, and remdesivir (both targeting RNA-dependent RNA polymerase—RdRp) [27,28]. However, as SARS-CoV-2 continues to spread, novel mutations are detected in its genome, resulting in the emergence of variants that have the potential to evade immunity against SARS-CoV-2, thus making existing treatments no longer effective [29,30,31]. This highlights the importance of having an arsenal of antiviral drugs against SARS-CoV-2 that target the different stages of its replicative cycle.

A useful tool for discovering therapeutic targets and drug candidates is computational chemistry. It helps us to understand protein–ligand interactions, using software to explore and calculate molecular properties [32]. For instance, computer-aided drug design (CADD) identified HIV drugs saquinavir and indinavir, which are approved for use in patients. Similarly, computational methods have aided in COVID-19 drug discovery [33,34,35,36].

## 2. Methods

**Figure 1:**
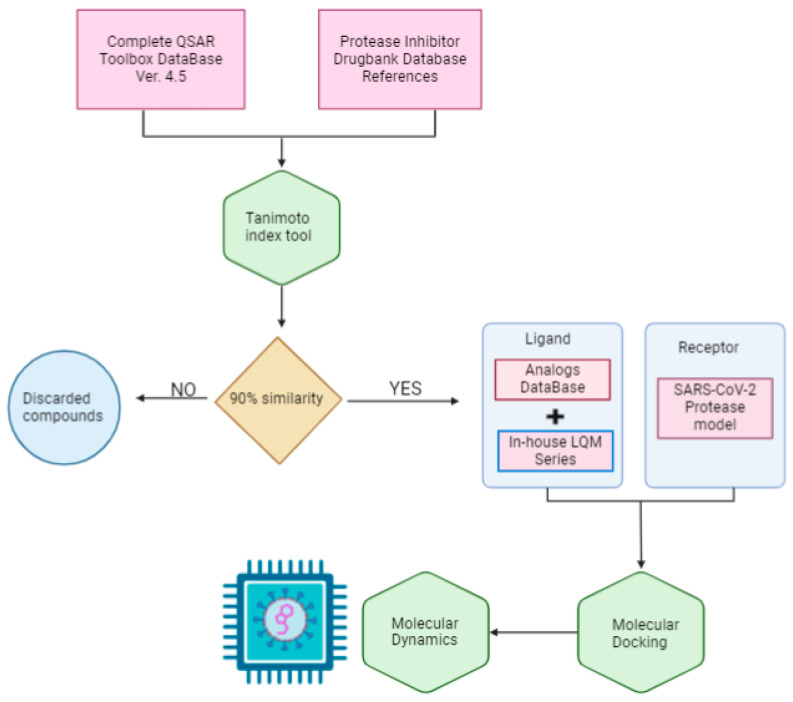
General workflow diagram.

The scope of this research is to identify potential hit molecules and propose a therapeutic strategy aimed at inhibiting the major protease of SARS-CoV-2. Figure 1 shows the computational approach taken in this work to screen and evaluate two sources of molecular structure data: the public QSAR Toolbox database and our internal database. Our in-house collection of chemical compounds has been compiled over 30 years of research and currently contains 250 compounds spanning a wide range of structures, functional groups, and activities, including antiviral, antineoplastic, antihypertensive, antiparasitic, and other properties [37,38].

The QSAR Toolbox database provided access to a wide variety of compounds. From this database, molecules were selected if they had a similarity of more than 90% to previously reported protease inhibitors. The Tanimoto coefficient was used as the decision criterion [39].

To identify these reference molecules, a broad search for protease inhibitors of any type was conducted in the DrugBank database. A total of 25 molecules were obtained and used as reference compounds to calculate the respective similarity coefficients. The resulting compounds were added to the molecules in our internal database to form the joint database.

Although the compounds in the LQM series had been previously studied in different diseases, it is not possible to encompass this research within the traditional concept of drug repositioning, since the QSAR Toolbox database is not specifically made up of molecules tested as drugs. It is important to clarify that the current research serves primarily as a screening process to identify potential hit molecules within this joint database. This approach has the potential to significantly accelerate the discovery of promising candidates for the development of effective drugs against COVID-19.

## 3. Results and Analysis

### 3.1. M^pro^ Structure Selection

SARS-CoV-2 M^pro^ is an important biological and pharmacological target [40]. Its structural integrity remains remarkably conserved across various coronavirus proteins owing to the high degree of conservation in its amino acid composition. In other words, its protein structure shows minimal mutations, resulting in a consistent conformational behavior [41].

**Figure 2:**
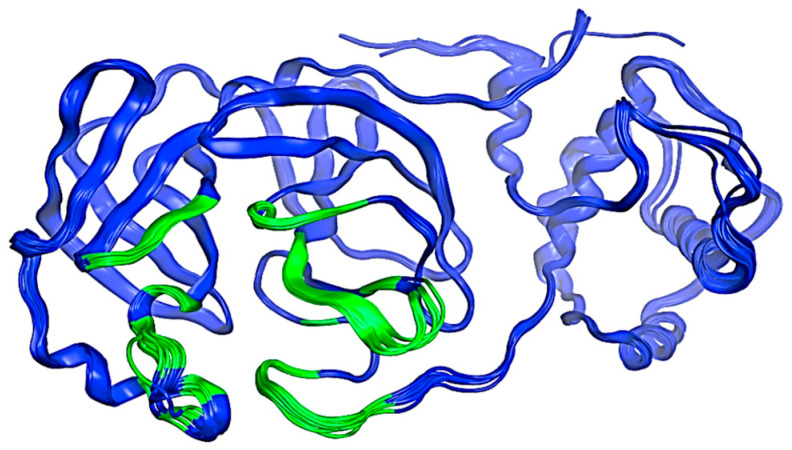
Structure of the SARS-CoV-2 M^pro^ protein. The possible interaction sites are colored green, and the rest of the protein is blue. Image created in the Molecular Operating Environment, MOE 2022.02.

An initial search was conducted within the PDB (Protein Data Bank), where several structures of M^pro^ were found. These structures are identified by the following codes: 7TOB, 5R81, 6R7Y, 6R83, 5R7Z, 5R82, 5R84, 6LU7, 6M03, 6Y2F, 6Y2G, 6Y8E, and 6YB7. All structures were aligned and superposed and the resulting model is illustrated in Figure 2. This preliminary exploration serves as a focal point for our structure selection. This is based on its status as one of the most up-to-date structures available in the PDB database compared to other proteins at the time [42]. The selection criteria for docking, virtual screening, and molecular dynamics simulations are discussed below.

**Figure 3:**
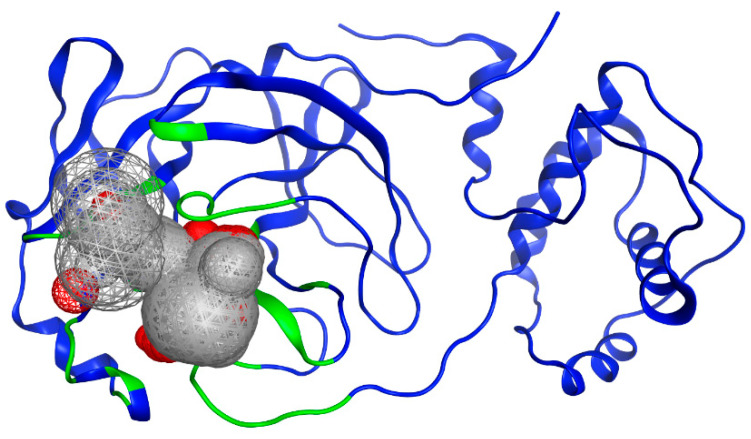
Potential binding site evaluated by SiteFinder. The size of the sphere represents how tightly packed the atoms are in the receptor; the larger the volume, the more accessible the atoms become. Spheres are colored by hydrophobic (gray) and hydrophilic (red). Image generated in MOE, 2022.02.

To identify potential active sites within the protein, the MOE SiteFinder tool was used [43]. This tool allows the detection of potential active sites in a receptor from its three-dimensional coordinates. It is a geometric method that considers the accessibility of the receptor atoms and a classification by chemical type. It excludes sites exposed to the solvent and classifies the different regions as hydrophobic or hydrophilic. Hydrophobic regions are represented as gray spheres, while hydrophilic regions are represented as red spheres. In Figure 3, the SARS-CoV-2 protein is presented along with the sites showing the highest interaction with the ligands. It is noteworthy that the amino acids His41 and Cys145 form a catalytic dyad [44], making them crucial elements for the subsequent experimental steps.

To identify potential protein binding sites or targets, ProteinsPlus, an online platform developed by the University of Hamburg, was utilized [45]. This platform primarily focuses on structure-based modeling, with an emphasis on protein-ligand interactions. Its functionalities include predicting protonation and tautomerization, categorizing various interactions in protein-protein complexes, and predicting binding sites and assessing their druggability, among other tools [45].

The approach is based on the concept of “druggability”, and the result of this assessment is a score that evaluates the feasibility of modifying a target using a small-molecule drug. This evaluation is crucial in the progression of a drug discovery project, transitioning from the computational “hit to lead” phase [46]. The druggability is used in drug discovery to elucidate a biological target, and it can be predicted with an affinity for a drug.

We applied the DoGSiteScorer tool, which is specialized in detecting binding sites, relying solely on the three-dimensional structure of the protein to deduce the overall properties of the pocket, including size, shape, and key characteristics. This tool generates a druggability score for each pocket, which is calculated based on a combination of three descriptors: volume, surface, and DrugScore. A higher score on this last parameter indicates a pocket with greater potential and favorability to be the target of a drug. In this context, it is desirable to have values that meet an acceptable threshold. These values range from 0.5 to 1.0, and those closer to 1.0 indicate pockets with strong druggability potential [47].

Table 1 shows the volume, surface, and DrugScore values, allowing us to identify the M^pro^ structure that is the most suitable for drug targeting based on these three values, thus advancing us to the next phase of this study.

For each SARS-CoV-2 main protease protein structure, between seven and nine potential binding pockets were identified. According to the results presented in Table 1, the 7TOB structure exhibits a druggability value of 0.81, positioning it as the candidate with the highest druggability potential. The 7TOB model corresponds to the Omicron variant 1.1.529, which is characterized by a highly conserved protein among the M^pro^ variants [41]. Therefore, we decided to select this protein to advance to the next phase of our project. Virtual screening (VS) studies have been conducted on this main protease structure to identify natural compounds as inhibitors, like theaflavin and ginkgetin [48].

**Figure 4:**
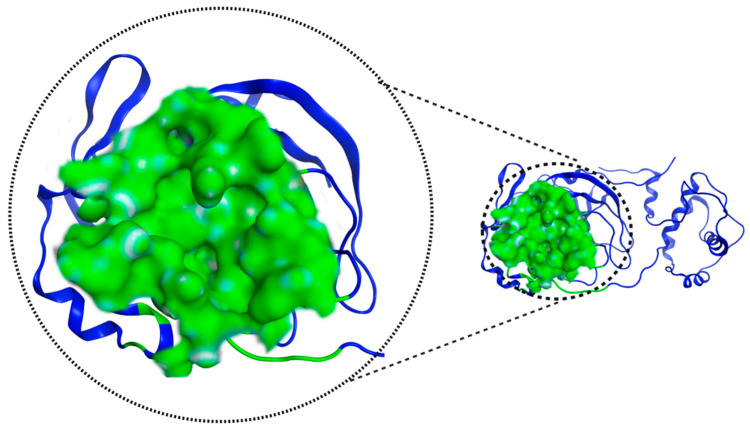
M^pro^ 7TOB potential interaction site, in green, defined by “Proteins*Plus*” online server.

Figure 4 shows the pocket where the catalytic dyad is located and presents an important feature of 7TOB: a volume of 673.22 Å^3^ and a surface area of 790.0 Å^2^.

**Figure 5:**
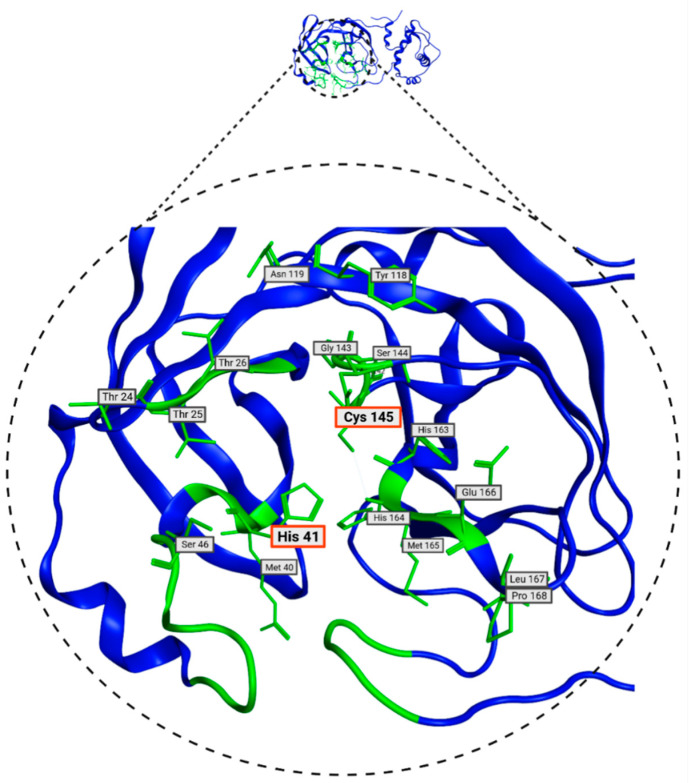
Consensus amino acids belonging to M^pro^ 7TOB site identified with DoGSiteScorer and SiteFinder from MOE 2022 tools.

In Table 2, the residues defined by Proteins*Plus* (DrugScore), which match with the SiteFinder process, are presented. These residues are the most likely to interact with molecules from the database and will be used in molecular docking. These residues are visualized in Figure 5 and play a crucial role in molecular docking processes. Some of the amino acids in the M^pro^ protein have already been identified as relevant in the literature. These are the His41 and Cys145 residues, which, as mentioned above, form a catalytic dyad and can inhibit protein activity [22]. Given the presence of these two residues, they prove to be a crucial part of inhibitor design [44].

**Figure 6:**
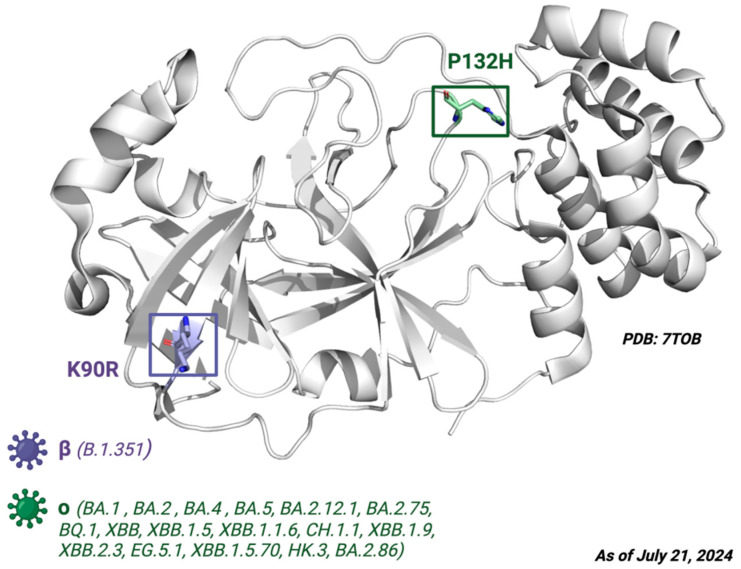
The crystal structure of M^pro^. Depiction of the M^pro^ amino acid substitutions in the SARS-CoV-2 variants of concern. The purple residue represents the K90R substitution present in the beta B.1.351 variant, whereas the green residue represents the P132H substitution present in the Omicron variants BA.1, BA.2, BA.4, BA.5, BA.2.12.1, BA.2.75, BQ.1, and XBB. Defining amino acid changes are those that appear at the phylogenetic root of a variant. Figure made with BioRender with a purchased license.

Next, the mutations defining the variants of interest (VOIs) such as B.1.1.7, B.1.351, P, B.1.617.2, BA.1, BA.2, BA.4, BA.5, BA.2.12.1, BA.2.75, BQ.1, XB, XBB.1.5, XBB.1.1.6, CH.1.1, XBB.1.9, XBB.2.3, EG.5.1, XBB.1.5.70, HK.3, and BA.2.86 were examined. ORF1ab substitutions shown in the CoVariants online platform [49] were converted to their respective amino acid sequence changes in Nsp5, then BLAST (Basic Local Alignment Search Tool, NIH) was used to align the polyprotein 1ab sequences. From this analysis, none of the VOIs had mutations in the target sites of the M^pro^ ligand. The only defining amino acid substitutions found were K90R for the beta variant B.1.351 and P132H for the Omicron variants BA.1, BA.2, BA.4, BA.5, BA.2.12.1, BA.2.75, BQ.1, XBB, XBB.1.5, XBB.1.1.6, CH.1.1, XBB.1.9, XBB.2.3, EG.5.1, XBB.1.5.70, HK.3, and BA.2.86 (Figure 6).

### 3.2. Selection of Potential Inhibitors of SARS-CoV-2 M^pro^

Once the M^pro^ structure and its corresponding binding site were defined, we proceeded to screen the quantitative structure–activity relationship (QSAR) Toolbox molecular database (QSAR Analytics S.A., Mexico), which contains 311,750 substances with various biological activities. We filtered the database using molecular similarity virtual screening using the Tanimoto coefficient as the decision criterion. We used twenty-five chemical structures of commercial antivirals previously identified as protease inhibitors, obtained from DrugBank, as references to determine compounds with high similarity with the QSAR Toolbox database, as shown in Table 3.

Atom-centered fragments together with saturated and aromatic cycles and incident pi bond (π bond) similarity variants were employed due to the structural characteristics of the antiviral compounds. We exclusively selected chemical structures that exhibited a similarity equal to or greater than 90% with respect to each of the antivirals used as references, resulting in a total of 2565 identified molecules. The selected molecules, sourced from DrugBank, share the common feature of being protease inhibitors and were considered due to their potential impact on the SARS-CoV-2 M^pro^ protein. As a result, 2565 molecules were obtained, as shown in Table 3.

We then proceeded to filter the compounds from our joint database (public + in-house) by virtual screening. The three-dimensional model of the M^pro^ 7TOB protein of SARS-CoV-2 was used as the receptor and the total of 2565 (QSAR Toolbox) and 250 (LQM series) compounds as ligands.

Virtual screening was performed in duplicate using two docking engines (AutoDock Vina and MOE) as consensus to compare the trend of the calculated scores [43,74,75]. Ensitrelvir (S-217622) and Atazanavir (BMS-232632) molecules were also included in the screening as reference ligands to compare the binding affinity score with the molecules studied here as these ligands have shown in vitro and in vivo activity and are even in clinical trial phases [76].

**Figure 7:**
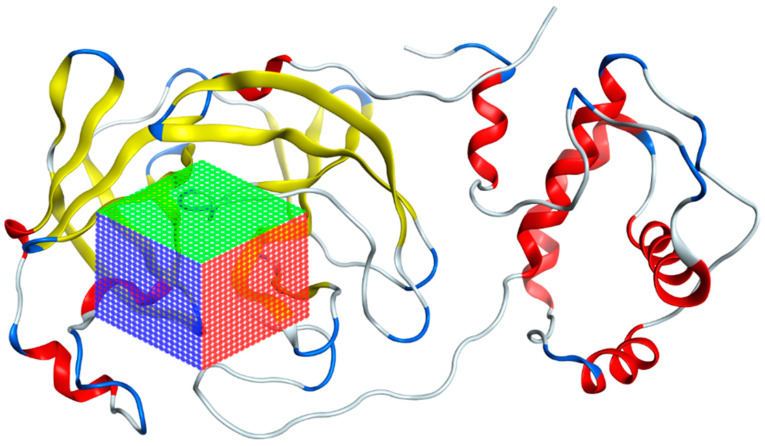
Grid of the 7TOB protein of SARS-CoV-2 in which the area where interactions with ligands were calculated is presented.

The preparation of AutoDock Vina input files was as follows: Using the AutoDockTools (ADT), the atomic coordinates of the SARS-CoV-2 main protease model were read from the PDB 7TOB file. The corresponding protein protonation was performed; then, the non-polar hydrogens were merged and the corresponding atomic partial Kollman charges were added. The receptor–ligand interaction zone was defined to include the amino acids identified above, generating a search box with the following dimensions: 25.50 Å × 28.05 Å × 25.50 Å (x, y, z) with the center located at an average distance from the atoms of the catalytic dyad (Cys145 and His41). Figure 7 illustrates the search box used for virtual screening. To prepare the ligand files, the PyRx tool was used to convert our compounds to the *pdbqt* format required for use in AutoDock Vina [77].

The MOE protocol was as follows: the alpha triangle matching algorithm was used to calculate initial poses for each molecule and, using the London dG scoring function, the 100 poses were obtained. These poses were refined and optimized, keeping the receptor rigid and evaluating with the GBVI/WSA dG scoring function, resulting in a total of 50 pose results for each ligand.

As a result of this filtering process, 22 compounds from the QSAR Toolbox database and 10 compounds from the LQM series were identified and are presented in the Table 4. The score values for each of the ligands obtained by the different docking engines are presented. The ligands with the most favorable interaction energy among the 32 compounds are highlighted: M^pro^L6 and LQM 778.

**Figure 8:**
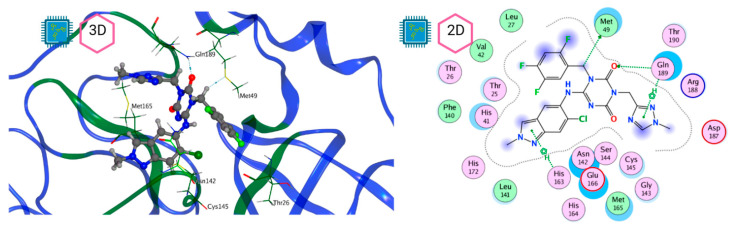
M^pro^–Ensitrelvir complex active site. The direct protein–ligand interactions are represented in the dotted line. Three-dimensional (**left**) and two-dimensional (**right**) representations.

**Figure 9:**
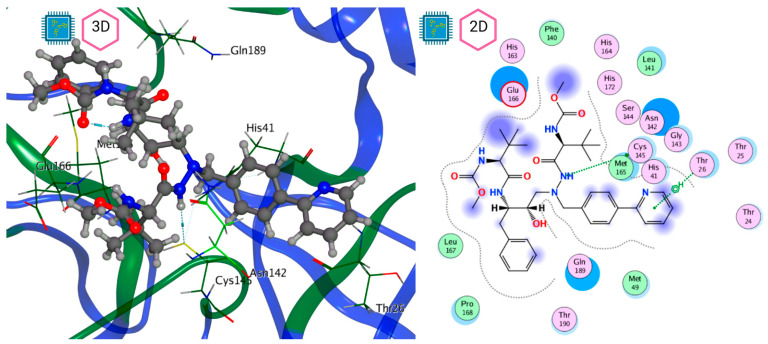
M^pro^–Atazanavir complex active site. The direct protein–ligand interactions are represented in the dotted line. Three-dimensional (**left**) and two-dimensional (**right**) representations.

**Figure 10:**
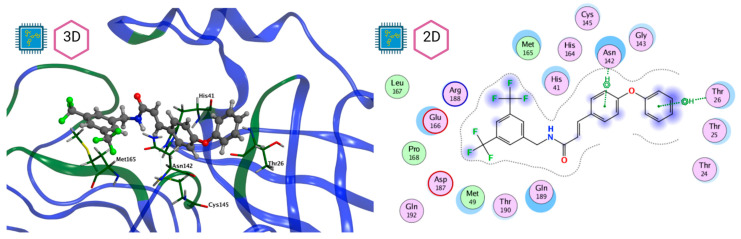
M^pro^–LQM 778 complex active site. The direct protein–ligand interactions are represented in the dotted line. Three-dimensional (**left**) and two-dimensional (**right**) representations.

Figure 8 and Figure 9 show the active site of M^pro^ complexes with Ensitrelvir (S-217622) and Atazanavir (BMS-232632) respectively. Interactions as a hydrogen bond with Thr-26 phenoxy group (H–-π interaction) and Asn 142 amide sidechain also appear in the complex with the compound LQM 778. Figure 10 shows the active site of the complex formed with the ligand LQM 778. Although some nearby amino acids do not form direct interactions with the ligand, there is geometric and hydrophilic complementarity.

Three molecular dynamics simulations of systems involving the M^pro^ protein were performed: one simulation of the protein without the ligand, called apo-form, and two simulations with M^pro^ complexes, one with the ligand M^pro^L6 and the other with LQM 778.

The systems for molecular dynamics simulations were prepared using the MOE 2022.02. software. Starting from the representative pose for each system, the system was solvated in a cubic water box at periodic conditions (P1, 95 Å per side) and Na^+^ and Cl^-^ counterions were added. Once solvated, a structural minimization of the whole system was performed. The input files were generated to run the molecular dynamics simulations in NAMD 2.13 software [78] using the Amber 14 force field, and the following protocol was established: first, a gradual heating stage from 0 to 300 K for 1 ns, followed by an equilibrium stage at 300 K and 1 atm pressure for 4 ns, to finally generate a 100 ns production stage where the pressure and temperature conditions were 300 K and 1 atm. For the molecular dynamic simulations, we used a cutoff of 12 and a time step of 0.002 fs.

**Figure 11:**
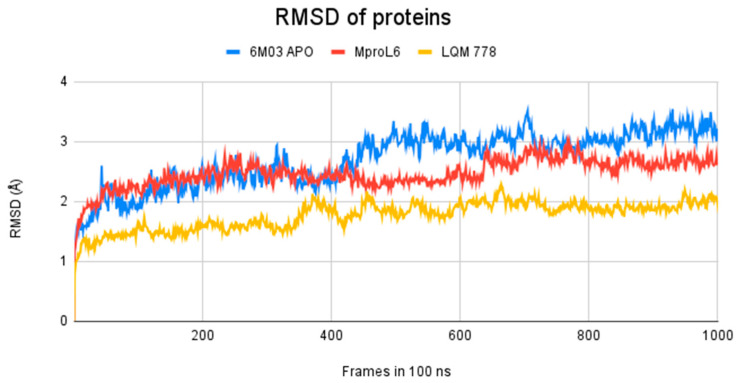
RMSD of M^pro^ systems: apo-form (in blue) and with the ligands M^pro^L6 (in red) and LQM 778 (in yellow).

**Figure 12:**
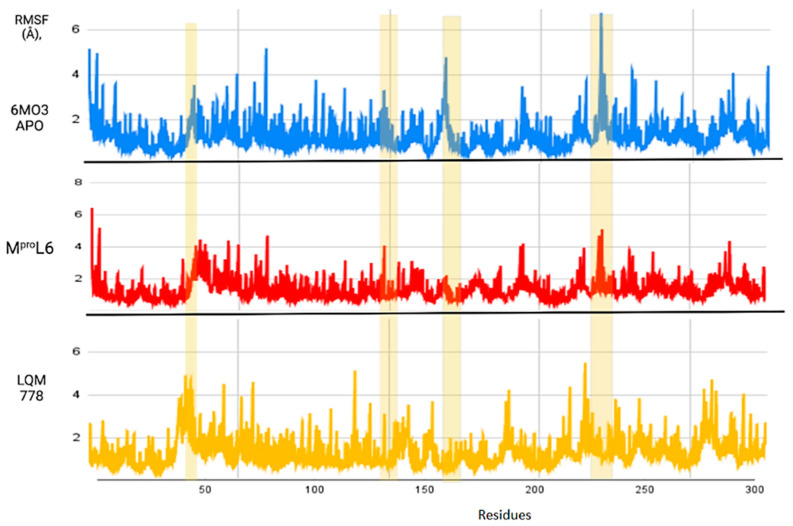
RMSF of the M^pro^ protein in its apo-form in blue, compared to the ligands M^pro^L6 in red and LQM 778 in purple.

**Figure 13:**
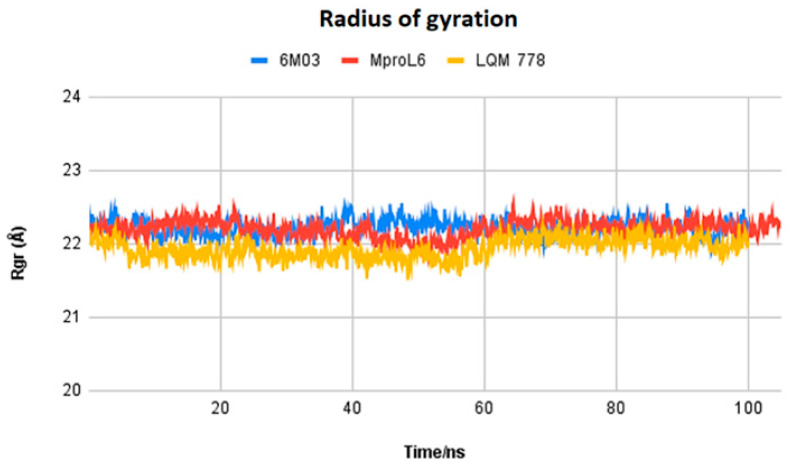
Radius of gyration of the ligands M^pro^L6 and LQM 778 and the apo-protein M^pro^.

After completing the molecular dynamics simulations, the following statistical parameters were calculated for each of these systems: RMSD (root mean square deviation) (Figure 11), RMSF (root mean square fluctuation) (Figure 12), and ROG (radius of gyration) (Figure 13).

The results obtained after calculations reveal that the two systems with ligands have a lower fluctuation in the RMSD values. It is observed that the system in its apo-form reaches a maximum distance of 3.5 Å, in contrast to the system with the M^pro^L6 ligand, which reaches a maximum distance of 3.1 Å. Finally, the system with the LQM 778 ligand has a maximum distance of 2.3 Å, suggesting that the complexes are more stable. Both ligands could maintain protein stability once they bind to the pocket.

Figure 12 shows the RMSF results, which allow us to identify the flexibility of the protein throughout the simulation, as shown in horizontal columns. The first two yellow columns indicate the region where the His41 and Cys145 residues are located. In addition, other areas are identified, such as that near residue 150, where the apo-protein showed an increase in its RMSF value, suggesting that once the ligands interact with the protein, the fluctuation of this region decreases. Finally, there is a noticeable fluctuation in the region near position 250; although it is not a significant fluctuation, it represents a distance greater than 6 Å in the apo-protein, which decreases in the plots with ligands. Therefore, it can be suggested that the protein remains in the states with more favorable stability and does not undergo deformations in its structure.

Figure 13 displays the radius of gyration of the systems: M^pro^L6 ligand in red, LQM 778 ligand in purple, and the SARS-CoV-2 M^pro^ protein in blue in its apo-form. Nonhomogeneous behavior among the structures can be observed at the beginning of the simulation and up to 50 ns. However, starting at 60 ns, the proteins maintain homogeneous behavior, indicating the stability of the system.

From the results obtained thus far, based on this analysis, it can be suggested that our identified candidate drugs could potentially be effective against circulating SARS-CoV-2 variants. Currently, the antiviral activity of these compounds is being evaluated in vitro in the laboratory of Dr. L. Abrahamyan (University of Montreal, Quebec, Canada).

## 4. Conclusions

In this study, our group investigated one of the key molecular targets of SARS-CoV-2, the protease M^pro^, of which, the PDB model 7TOB crystal structure served as a central target in our in silico molecular repositioning studies with diverse biological activities. Using a virtual screening (VS) approach, we applied the concept of repositioning using two databases, one external and one generated by our group. This approach allowed us to identify molecules with high potential as promising M^pro^ inhibitors.

The computational studies consisted of estimating the activity of a selection of molecules—a joint database composed of those from an in-house LQM series and the QSAR Toolbox database—against M^pro^ SARS-CoV-2. Two docking software were used to calculate an interaction score and, by consensus of the results, the molecules were ranked by best energy value. As a result of this process, we identified a total of thirty-two promising candidates, twenty-two from the external database and ten from our own collection of the LQM 700 series. Notably, the most relevant interactions with the protein residues His41 and Cys145 remained constant in all candidates, validating the robustness of our findings. Thus, by a comprehensive analysis of the in silico results, we determined that two molecules have significant potential as antiviral agents against SARS-CoV-2: M^pro^L6 and LQM 778. These findings were supported by an assessment of computational integrity, including RMSD, RMSF, and radius of gyration, increasing confidence in their potential candidacy for future research and development. Furthermore, given that SARS-CoV-2 is in constant genetic evolution, looking at the latest variants revealed that the only significant amino acid substitutions found in the latest variants are in the K90R and P132H positions. This may suggest that our inhibitors identified in this study could target the M^pro^ of the latest SARS-CoV-2 variants, making them of great interest, especially in the context of the emergence of novel variants. With further in vitro and in vivo research, our results in this article can give rise to an arsenal of molecules against COVID-19.

## Figures and Tables

**Table 1:** DoGSiteScorer values for different M^pro^ models.

*Protein*	*Volume* (Å^3^)	*Surface* (Å^2^)	*Drug Score (Druggability)*
**7TOB**	673.33	790.00	0.81
**5R81**	623.55	648.20	0.71
**5R7Y**	618.69	718.99	0.77
**5R83**	623.80	701.32	0.77
**5R7Z**	623.89	669.87	0.78
**5R82**	628.54	764.97	0.72
**5R84**	588.10	717.52	0.74
**6LU7**	398.59	629.05	0.73
**6M03**	523.87	747.87	0.78
**6Y2F**	600.64	672.26	0.70
**6Y2G.A**	672.29	756.12	0.80
**6Y2G.B**	660.16	670.12	0.73
**6Y8E**	589.38	775.76	0.79
**6YB7**	529.98	682.30	0.76

**Table 2:** List of amino acids that make up the pocket of SARS-CoV-2 M^pro^ (7TOB).

**Thr26, Leu27, His41, Ser46, Met49, Tyr118, Asn119, Phe140, Leu141, Asn142, Gly143, Ser144, Cys145, His163, His164, Met165, Glu166, Leu167, Pro168, His172, Gln189**

**Table 3:** Count of compounds filtered by Tanimoto coefficient and their protease inhibitor reference.

Protease Inhibitor	Number of Analogs	Activity	Target/Uniprot ID	References
ASC09 (TMC-310911)	4	Protease inhibitor (PI) with activity against a variety of HIV-1 strains including multi-PI-resistant strains	Gag-Pol polyprotein/P03366 HIV-1 protease/O90777	Dierynck et al. (2011) [50]
Nelfinavir	16	Used in the treatment of HIV infection, inhibits viral proteinase enzyme which prevents cleavage of the gag-pol polyprotein, resulting in noninfectious, immature viral particles.	HIV-1 protease/O90777	Kaldor et al. (1997) [51]
Baicalein	276	Baicalein is under investigation in clinical trials in the treatment of healthy adults with influenza fever.	Lactoylglutathione lyase/Q04760 Tumor necrosis factor/P01375 Xanthine dehydrogenase-oxidase/P47989 L-selectin/P14151 Prolyl endopeptidase/P48147 Polyunsaturated fatty acid 5-lipoxygenase/P09917	Islam et al. (2021) [52]
Remdesivir	3	Nucleoside analog used to treat RNA virus infections by inhibiting the RNA polymerase (RdRp) enzyme complex for genomic replication.	Replicase polyprotein 1ab/SARS-CoV: P0C6X7 SARS-CoV-2: P0DTD1 RNA-directed RNA polymerase L/Q05318	Sheahan et al. (2020) [53]
Bromhexine	305	Mucolytic agent, derived from the Adhatoda vasica plant; used for a variety of respiratory conditions associated with increased mucus secretion.	Transmembrane protease serine 2/O15393 Angiotensin-converting enzyme 2/Q9BYF1	Zanasi et al. (2017) [54]
Ritonavir	61	HIV protease inhibitor used in combination with other antiviral agents for the treatment of HIV infection.	Gag-Pol polyprotein/P03366	Hull et al. (2011) [55]
Boceprevir	77	NS3/4A protease inhibitor for hepatitis C virus, used in combination with other medications to treat chronic hepatitis C genotype 1 infection. It is not indicated for use as monotherapy.	Genome polyprotein/P26664	Kiser et al. (2013) [56]
Saquinavir	56	HIV protease inhibitor used in combination with other antiretroviral agents for the treatment of HIV-1 in patients with advanced immunodeficiency.	Gag-Pol polyprotein/P03366	Kupferschmidt et al. (1998) [57]
Camostat	554	Serine protease inhibitor approved in Japan for the treatment of chronic pancreatitis.	Metal ion binding/P07477	Kitamura et al. (2012) [58]
Simeprevir	2	Direct-acting antiviral agent that inhibits the HCV NS3/4A protease; used to treat chronic hepatitis C virus (HCV) infection in adults with HCV genotype 1 or 4.	Genome polyprotein/P26664	Raboisson et al. (2008) [59] Bafna et al. (2011) [60]
Cyanidin 3-glucoside	56	Anthocyanidin phytochemical and metabolite found in several plants such as angiosperms; also produced by *Saccharomyces cerevisiae.*	No information about a specific protease. The ability to inhibit the activity of cyclooxygenase enzymes has been demonstrated.	Islam et al. (2021) [52]
Vaniprevir	2	In clinical trials for the treatment and diagnosis of Hepatitis C, including chronic forms and genotype 1 infections.	Gag-Pol polyprotein/P03366	Bafna et al. (2011) [60]
Chrysphanol 8′(6-galloylgluside	16	Anthraquinone derivative isolated from rhubarb; it has an inhibitory effect on platelet aggregation induced by collagen and thrombin.	No information about a specific protease.	Alamri et al. (2020) [61]
Umifenovir	193	Dual-function antiviral and host-targeting agent used for the treatment and prevention of influenza and other respiratory viruses.	No information about a specific protease.	Lu et al. (2020) [62]
Darunavir	62	HIV protease inhibitor employed in treating HIV infection, particularly in patients with a history of previous antiretroviral therapies.	Aspartic-type endopeptidase activity UniProt: Q72874	Purohit et al. (2009) [63]
Tipranavir	26	Protease inhibitor used to treat HIV-1 that is resistant to multiple other protease inhibitors.	Aspartic-type endopeptidase activity UniProt: Q72874	Doyon et al. (2005) [64]
Gc376	155	Direct-acting antiviral for coronaviruses such as MERS-CoV, feline, ferret, and mink.	Replicase polyprotein 1ab SARS CoV II: P0DTD1 Feline: Uniprot Q98VG9	Ye et al. (2020) [65]
Triazavirin	33	Influenza A and B infections	Triazavirin is a guanosine nucleotide analog that inhibits RNA synthesis.	Kiselev et al. (2012) [66]
Isocodonocarpine	110	Phytochemical identified in silico for its binding to SARS-CoV-2 papain.	No information about a specific protease	Khatib et al. (2016) [67]
Withanolide A	19	Phytochemical that exhibits stronger binding with M^pro^ compared to hydroxychloroquine.	No information about a specific protease	Srivastava et al. (2022) [68]
Iso-mulbel-rochromene	13	Phytochemical inhibitor identified for inhibition of 3CLpro protease.	No information about a specific protease	Tao et al. (2023) [69]
α-Ketoamide- 11r	83	Peptidomimetic compound designed for antiviral activity against M^pro^ coronavirus and the enterovirus 3C protease.	Replicase polyprotein 1ab/P0C6X7	Islam et al. (2021) [52] Zhang et al. (2020) [70]
Lopinavir	101	HIV-1 protease inhibitor used in combination with ritonavir for the treatment of HIV infection.	Aspartic-type endopeptidase activity/Q72874	Sheahan et al. (2020) [53]
x77	174	Standard inhibitor identified for the inhibition of the 3CLpro protease	No information about a specific protease	Sharma et al. (2023) [71]
Nafamostat	168	Used in trials studying the prevention of liver transplantation and postreperfusion syndrome. Anticoagulant therapy for patients undergoing continuous renal replacement therapy.	Tumor necrosis factor/P01375 Prothrombin/P00734 Coagulation factor X/P00742 Serine protease 1/P07477 Kallikrein-1/P06870 Intercellular adhesion molecule 1/P05362	Hoffmann et al. (2020) [72] Yamamoto et al. (2016) [73]
Total analogs	2565			

**Table 4:** Predicted score values by AutoDock Vina and MOE for 32 selected compounds.

Ligand	Structure	AutoDock Vina Score (kcal/mol)	MOE Score (kcal/mol)	Residues of the M^pro^ That Interact with the Ligands
Ensitrelvir (S-217622)	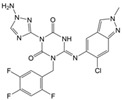	−7.3	−8.57	Met49, Cys145, His163, Gln189
Atazanavir (BMS-232632)	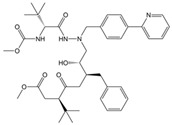	−6.3	−9.48	Thr26, Asn142, Cys145, Glu166
**M^pro^L6**	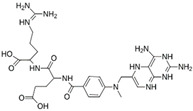	**−7.8**	**−8.12**	**Asn142, Thr25, Glu166, Cys145**
M^pro^L13	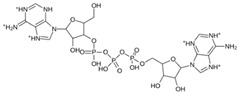	−7.4	−7.22	Asn142, Glu166, Cys145, Thr25, Met49
M^pro^L16	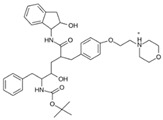	−6.8	−7.81	Asn142, Glu166, Thr25
M^pro^L4	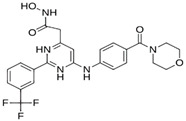	−7.5	−6.84	Asn142, Thr25, Glu166, Gln143
M^pro^L11	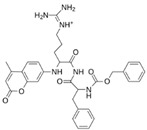	−6.7	−7.47	Asn142, Glu166, Cys145, His41, Thr25
M^pro^L21	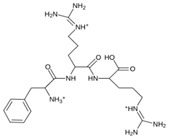	−6.8	−7.3	Asn142, Glu166, Cys145, Thr26, Met49
M^pro^L5	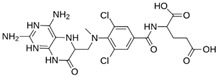	−7.2	−6.81	Gly143, Thr25, Glu166, Cys145
M^pro^L7	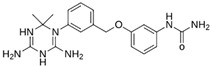	−7.4	−6.56	Thr25, Asn142, Glu166, Met165
M^pro^L8	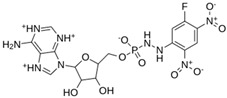	−7	−6.92	Asn142, Cys145, Glu166, Met165, Thr25
M^pro^L17	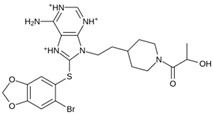	−6.4	−7.11	Asn142, Cys145, Glu166, Thr25
M^pro^L15	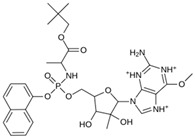	−6.6	−6.75	Asn142, Cys145, Thr25
M^pro^L22	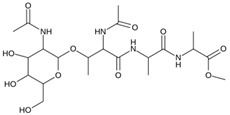	−6.1	−7.24	Asn142, Cys145, Glu166, Thr25, Met49
M^pro^L18	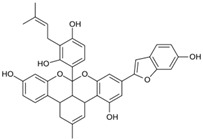	−6.4	−6.55	Asn142, Ser46, Thr25, Glu166
M^pro^L2	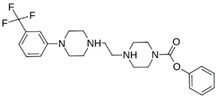	−6.2	−6.65	Asn142, Cys145, Thr25, His41, Met49, Glu166
M^pro^L10	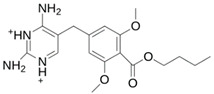	−6.3	−6.5	Asn142, Glu166, Thr25, Met165
M^pro^L14	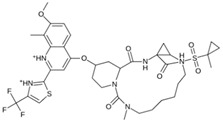	−5.6	−7.05	Asn142, Thr25, Glu166, Cys145
M^pro^L20	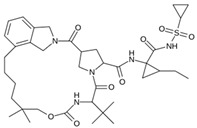	−5.5	−7.13	Asn142, Thr25, Glu166, Met49, Cys145
M^pro^L19	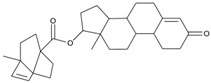	−6.1	−6.3	Asn142, Thr25, Glu166
M^pro^L12	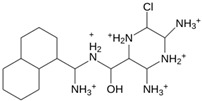	−6.8	−4.91	Asn142, Glu166, Cys145, Met49, His41, Thr25
M^pro^L1	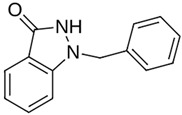	−5.6	−5.29	Asn142, Cys145, Gly143
M^pro^L9	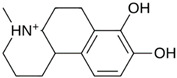	−5.7	−4.92	Asn142, Leu141, His41, Glu166
M^pro^L3	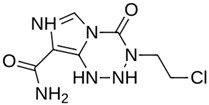	−5.1	−5.17	Cys145, Met165, Glu166, Arg188, Gln189
**LQM 778**	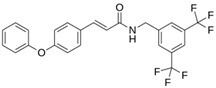	**−8.7**	**−7.2**	**His41, Cys145, Met165**
LQM 769	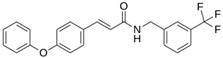	−8.3	−6.8	Asn142, Cys145, Glu166
LQM 794	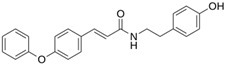	−7.2	−7.2	Thr25, Asn142, Glu 166, Met165
LQM 764	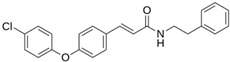	−6.9	−6	Asn142, Cys145, Glu166
LQM 796	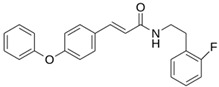	−6.9	−5.8	Cys145, Thr25, His41
LQM 797	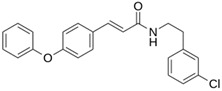	−6.9	−5.9	Asn142, Glu166, Met165
LQM 795	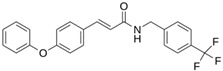	−6.9	−6.2	His41, Met49, Glu169
LQM 721	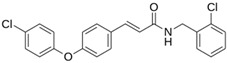	−6.7	−5.2	Glu169, Met49
LQM 755	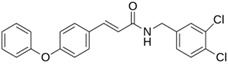	−6.6	−5.6	Asn142, Glu166, Thr25
LQM 798	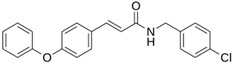	−6.4	−5.3	Ser46, Thr25, His41

## Data Availability

Derived data supporting the findings of this study are available from the author M.A.H.S on request.
